# Evaluation of *CYP2C8* and *CYP2C9* Polymorphisms in Neonates with Patent Ductus Arteriosus Treated with Ibuprofen or Indomethacin: A Retrospective Cohort Study

**DOI:** 10.3390/jcdd13010049

**Published:** 2026-01-15

**Authors:** Shaikha Jabor Alnaimi, Shimaa Aboelbaha, Ibrahim Safra, Mai Abdulla Al Qubaisi, Fouad Abounahia, Ahmed Al Farsi, Liji Cherian, Lizy Philip, Moza Alhail, Gulab Sher, Nader Al-Dewik

**Affiliations:** 1 Pharmacy Department, Women’s Wellness and Research Center, Hamad Medical Corporation, Doha 3050, Qatar; shimaakamel2378@gmail.com (S.A.); malhail2@hamad.qa (M.A.); 2 Neonatal Intensive Care Unit, Women’s Wellness and Research Center, Hamad Medical Corporation, Doha 3050, Qatar; isafra@hamad.qa (I.S.); malqubaisi@hamad.qa (M.A.A.Q.); fabounahia@hamad.qa (F.A.); ahmed.111@live.com (A.A.F.); 3 London Health Sciences Center, Western University, London, ON N6A 3K7, Canada; 4 Baby Clinic, Outpatient Department, Women’s Wellness and Research Center, Hamad Medical Corporation, Doha 3050, Qatar; lcherian4@hamad.qa (L.C.); ljohn1@hamad.qa (L.P.); 5 Research Department and Translational and Precision Medicine Research, Women’s Wellness and Research Center, Hamad Medical Corporation, Doha 3050, Qatar; gulab.sher@yahoo.com (G.S.); naldewik@hamad.qa (N.A.-D.)

**Keywords:** patent ductus arteriosus, pharmacogenomics, neonates, NSAIDs, translational research

## Abstract

The pharmacologic management of patent ductus arteriosus (PDA) presents a challenge to clinicians due to the interindividual variability in drug response to available medications. There is evidence that *CYP2C9* is associated with the response to PDA treatment; however, no data from the Middle East is available. This study aimed to investigate the association between *CYP2C8* and *CYP2C9* genetic polymorphisms and response to ibuprofen or indomethacin in neonates with PDA. We conducted a retrospective cohort study of neonates with a gestational age < 32 weeks and birthweight < 1500 g with PDA between 2019 and 2023. Eligible neonates were those diagnosed with PDA and treated with at least one course of ibuprofen or indomethacin. Genotyping was performed to identify four single-nucleotide polymorphisms (SNPs), namely CYP2C8*3 rs10509681, CYP2C9*2 rs1799853, *CYP2C9* rs2153628, and CYP2C9*3 rs1057910. Allele frequencies were compared between responders and non-responders, and non-genetic predictors were assessed using logistic regression. A total of 146 infants were identified. Of these, 86 were enrolled. Genetic analysis showed that the heterozygote genotype (TC) for the *CYP2C8* gene was the most common (45%), while wild-type alleles were predominant for *CYP2C9* variants. No significant differences in allele frequencies were found between responders and non-responders to the treatment (*p* > 0.05). In a secondary analysis, the need for multiple surfactant doses independently predicted poor response (aOR 0.244, 95% CI 0.086–0.693, *p* = 0.008), while extremely low birth weight showed a borderline association (aOR 0.281, 95% CI 0.062–1.268, *p* = 0.099). Carriers of CYP2C8*3 rs10509681, CYP2C9*2 rs1799853, *CYP2C9* rs2153628, and CYP2C9*3 rs1057910 were not associated with variations in response to NSAIDs.

## 1. Introduction

Pharmacogenomics (PGx) is defined as the study of interindividual variability in response to medications, attributed to differences in individuals’ genetic makeup [1]. It supports the shift in clinical care from a “one-size-fits-all” approach to a more personalized approach [2]. The adoption of genetics-guided pharmacotherapy is increasing in various fields, such as cardiology, oncology, and psychiatry [3]. However, its implementation in neonatology is limited. This is partially due to the lack of studies investigating the effect of genetic variants on clinical outcomes in this population. Furthermore, the progressive developmental ontogeny of the pharmacokinetic and pharmacodynamic pathways of neonates hinders the extrapolation of adult PGx data [4,5].

One of the areas that warrant PGx investigation is the management of patent ductus arteriosus (PDA). The incidence of PDA is 20–50% and is more common in preterm infants (<32 weeks) [6,7]. It is also associated with 3.5% mortality and long-term complications such as heart failure, infective endocarditis, pulmonary hypertension, chronic lung disease, and neurodevelopmental impairments [8,9,10]. The management of PDA includes observation, pharmacologic treatment, or surgical ligation. The pharmacologic mechanism for ductus closure involves inhibiting the effect of prostaglandins using non-steroidal anti-inflammatory drugs (NSAIDs), namely ibuprofen and indomethacin [11,12,13]. Clinical research reports that up to 40% of patients fail to respond to initial NSAID treatment [14,15] and that up to one-third require surgical ligation [16].

A study by Smith et al. suggested that this variation in response could be due to an underlying genetic mechanism [17]. Cytochrome P450 (CYP450) enzymes, including CYP2C9 and CYP2C8, are largely responsible for the metabolism of indomethacin and ibuprofen [18]. In adults, it has been reported that interindividual variability attributed to genetic polymorphisms in both enzymes contributes to alterations in NSAID pharmacokinetics [18,19]. In neonates, the effect of genetic variants is further complicated by maturation-dependent changes in gene expression [20], which warrants exploring the potential impact of genetic polymorphisms of CYP450 enzymes on the responsiveness in this population.

In the literature, there is inconsistent evidence of the effect of CYP450-encoding variants on the clinical outcomes of using NSAIDs for PDA. A previous study compared neonates who responded to indomethacin and those who required surgical ligation in terms of the *CYP2C9* single-nucleotide polymorphism (SNP). The study showed a higher likelihood of PDA closure in newborns who had the G allele of the rs2153628 variant when treated with indomethacin compared to those with other alleles (*p* < 0.05) [17]. Another study failed to find a statistically significant difference in CYP2C9*2 and *3 variants’ frequency between responders and non-responders in neonates (≤30 weeks) treated with ibuprofen [21].

Moreover, population-specific genetic differences may influence drug response patterns and clinical outcomes. However, to date, no studies have examined the association between CYP450 polymorphisms and NSAID response in neonates from the Middle East region. Therefore, this study aimed to assess the association of genetic polymorphisms in *CYP2C8* and *CYP2C9* with response to ibuprofen and indomethacin in neonates with PDA within a Middle Eastern population.

## 2. Methodology

### 2.1. Study Population and Data Collection

We conducted a retrospective study that included infants with gestational age < 32 weeks and birth weight < 1500 g with PDA who were discharged from the neonatal intensive care unit (NICU) between 1 January 2019 and 31 December 2023 at the Women’s Wellness and Research Center, Qatar. The management of PDA in our institution follows a local guideline. Pharmacologic treatment is indicated for hemodynamically significant PDA (hsPDA), which is primarily determined by echocardiographic assessment of the ductal diameter, pulmonary overflow, and evidence of systemic hypoperfusion. Hemodynamic significance was defined as a ductus diameter ≥ 1.5 mm with a pulsatile left-to-right shunt pattern, together with at least two markers of pulmonary overflow (left atrium-to-aortic root [LA/Ao] ratio ≥ 1.5, isovolumic relaxation time [IVRT] < 55 ms, left ventricular output > 300 mL/kg/min, or pulmonary artery end-diastolic flow ≥ 0.3 m/s) and evidence of systemic hypoperfusion, namely absent diastolic flow in the abdominal aorta and celiac trunk.

When pharmacologic treatment was indicated, ibuprofen was used as first-line therapy and administered intravenously or orally at an initial dose of 10 mg/kg, followed by two doses of 5 mg/kg given at 24 h intervals (3-dose course). A second course could be given when post-treatment echocardiography did not demonstrate interval ductal constriction. Indomethacin was administered intravenously in cases without echocardiographic improvement following ibuprofen, with a loading dose of 0.2 mg/kg followed by two doses of 0.1–0.2 mg/kg administered at 12–24 h intervals (total of 3 doses). Paracetamol was used (15 mg/kg intravenously every 6 h for 5–7 days) when ibuprofen or indomethacin was contraindicated or ineffective. Echocardiography was repeated after the completion of each treatment course to assess closure.

Eligible infants were those diagnosed with PDA by their first echocardiogram and treated with at least one course of ibuprofen or indomethacin. Exclusion criteria were the presence of congenital anomalies, bleeding disorders, and any infant with Do Not Resuscitate (DNR) status. In addition, infants with intrauterine growth restriction (IUGR) were excluded to reduce clinical heterogeneity, as IUGR is associated with altered cardiovascular physiology, including differences in ductal patency regulation that may influence treatment response and pharmacogenomic interpretation.

Parents of eligible infants were contacted, and written informed consent was obtained from at least one parent to allow prospective buccal sample collection and the use of de-identified electronic health record data. After enrollment, appointments were scheduled for buccal sample collection. Following this, clinical and laboratory outcome data were collected retrospectively from electronic health records. Data collected included demographic information, perinatal data, clinical management parameters, and laboratory variables. The primary analysis was the association between genetic polymorphisms and NSAID treatment response. Treatment response was assessed using the post-treatment echocardiography report, performed within ten days post-treatment. An infant was classified as a responder if the ductus was tiny or closing, and a non-responder if it remained moderate to large or needed surgical intervention. Secondary analysis was used to assess non-genetic predictors of treatment response. The reporting of this observational study followed the STROBE checklist (Supplementary Material).

### 2.2. Sample Collection

Buccal samples were collected from infants to perform genetic testing, by two trained nurses from the outpatient clinic. iClean CY-98000 (Huachenyang, Shenzhen, China) disposable flocked nylon nasopharyngeal swabs were utilized for this purpose [22]. Using moderate strength, swabs were inserted inside the oral cavity, placed against the inner wall mucosa of each cheek, and rotated from the inside to the outside for 10–15 s. In order to enhance the yield, two swabs were used for each infant, and they were both stored in the same tube [22]. Following collection, the swabs were broken off at the break mark and inserted into the sampling tubes. They were then labeled and transported to the Genetic Interim Translational Research Institute laboratory in Hamad Medical Corporation for DNA extraction.

### 2.3. DNA Extraction

Automated purification of DNA from buccal samples was performed using the Maxwell^®^ 16 DNA Purification Kits Protocol (Promega Corporation, Madison, WI, USA) [23]. Instructions for using the human buffy coat kit (Cat.# AS1010) (Promega Corporation, Madison, WI, USA) were followed throughout the extraction process. Before starting the procedure, swabs were removed from their original tubes and discarded using sterilized forceps. Then, tubes containing the genetic material dissolved in buffer were centrifuged for 20 min at 2000 *g* using a microcentrifuge. Following this step, each sample was divided into two parts, a supernatant and pellet, and the parts were handled as two new samples to ensure an adequate amount of DNA was available for genotyping. Then, Maxwell cartridges were prepared, and each sample was transferred to a separate cartridge. Samples were placed in well #1, and plungers were placed in well #7. Additionally, tubes containing elution buffer were prepared according to the number of samples being analyzed (one tube per sample), with each tube containing 200 µL of buffer. Finally, the cartridges and the elution tubes were transferred to the Maxwell^®^ 16 MDx Instrument (SEV) (Promega Corporation, Madison, WI, USA) platform, and they were placed in a known orientation to begin the purification run. Once the run was complete, the extracted DNA was transferred to new, properly labeled tubes and sent for quantity and quality assessments.

### 2.4. DNA Quantification

The assessment of the quality and quantity of the extracted DNA was performed using the NanoDrop 2000c spectrophotometer (Thermo Fisher Scientific Inc., Waltham, MA, USA) [24]. Initially, 1.5 µL of elution buffer was used to perform a blank measurement. Then, 1.5 µL of the extracted DNA was used to measure DNA quantity and quality for all samples. Readings of DNA concentration, with ratios of 260/280 and 260/230, were then recorded and used to assess the quality and quantity of the extracts. All steps of the procedure were performed following the instructions on the Nanodrop 2000/2000c Spectrophotometer V1.0 User Manual [24].

### 2.5. Single-Nucleotide Polymorphism Detection and Genotyping

SNP detection and genotyping were carried out using the Applied Biosystems Real-Time Polymerase Chain Reaction (RT-PCR) Quant Studio™ 12K Flex (Thermo Fisher Scientific Inc., USA) instrument. Four SNPs on genes known to affect the metabolism of NSAIDs were tested. The characteristics of the SNPs are shown in Table 1. All procedures were conducted according to instructions for 96-well plate reactions on the TaqMan SNP Genotyping Assay and TaqMan Drug Metabolism Genotyping Assay protocols [25,26]. Reaction mixes were prepared according to the volumes listed in Table 2. Each reaction contained 10 ng of genomic DNA; thus, 11.25 µL was extracted from each sample to ensure that each well contained 0.9 ng of DNA. Nuclease-free water was used in the dilution process; therefore, it was not added during plate preparation. The PCR conditions for each SNP are detailed in Table 3.

### 2.6. Statistical Analysis

Continuous variables are presented as mean ± standard deviation or as median and interquartile range (IQR), according to data distribution. Categorical variables are presented as frequencies and percentages. Baseline clinical and laboratory variables were compared between responders and non-responders using Student’s *t*-test and the Mann–Whitney U test for normally and non-normally distributed variables, respectively. Chi-square or Fisher’s exact test were used for categorical variables. Genotype distributions of the studied variants were tested for deviation from the Hardy–Weinberg equilibrium using the chi-square test, with *p* > 0.05 considered consistent with equilibrium. The primary analysis examined associations between genetic polymorphisms and treatment response, and frequencies were compared using Chi-square tests. Additionally, non-genetic predictors of response were assessed initially with a univariate logistic regression to identify potential predictors of treatment and variables with *p* < 0.05, in addition to gestational age, as a possible confounder. The results are presented as odds ratios with 95% confidence intervals. Based on the published data [17], an odds ratio (OR) of 2.4 was assumed. With a two-sided α of 0.05 and 80% power, the required sample size was calculated as 180 participants. Statistical significance was set at *p* < 0.05. All statistical analyses were performed using SPSS version 27.0 (IBM Corp., Armonk, NY, USA).

## 3. Results

**Figure 1:**
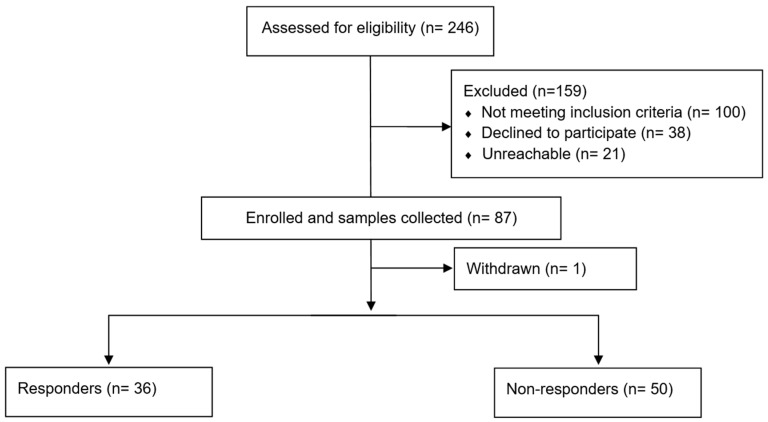
Flow diagram of participants.

Over the 4-year study period, a total of 146 infants who received an NSAID to treat PDA were identified. Among these, 86 were enrolled after obtaining signed, informed consent from at least one parent and underwent genetic analysis (Figure 1). Among these, 56% were males and 58% were of Middle Eastern descent. The median gestational age and birth weight were 25.5 (interquartile range (IQR) 2.8) weeks and 840 (IQR 253.8) grams, respectively. The majority were singleton pregnancies (70%), and around half were delivered via normal vaginal delivery (56%). Most infants required intubation at birth (81%), and surfactant was administered to 91% of infants. Among the 86 enrolled infants, 36 (42%) responded to drug therapy within the first 10 days following treatment and were defined as responders. Ibuprofen was given to all infants as the initial pharmacologic treatment for PDA, with a median of four doses, and only eight infants (9%) were given a trial of indomethacin following ibuprofen. Non-responders received significantly more total NSAID doses (median 6 vs. 3, *p* < 0.001). Of the 50 non-responders, 10 (20%) patients received subsequent paracetamol therapy, and 7 (14%) required surgical ligation. Ibuprofen was discontinued in six infants due to possible adverse reactions, including acute kidney injury and bowel distension.

The baseline characteristics are shown in Table 4. Responders and non-responders were similar in the majority of clinical characteristics but statistically differed (*p* < 0.05) in birthweight category and number of surfactant doses.

The frequency of genetic variants in the study population is presented in Table 5. All genetic variants were in Hardy–Weinberg equilibrium (*p* > 0.05), indicating no evidence of genotyping errors or population stratification. While the heterozygote genotype (TC) was the most prevalent in the study population (45%) for the *CYP2C8* gene, the wild-type allele constituted the most common genotype among the study participants for all variants on the *CYP2C9* gene. No statistically significant differences were observed among responders and non-responders in terms of the frequency of different alleles for all study variants (*p* > 0.05).

The results of the univariate regression are presented in Table 6. In univariate analysis, extremely low birth weight (ELBW) (OR 0.326, 95% CI 0.098–0.695, *p* = 0.007) and need for two or more surfactant doses (OR 0.261, 95% CI 0.084–0.625, *p* = 0.004) were significantly associated with poor treatment response. Gestational age (per week) showed no significant association with response (OR 1.165, 95% CI 0.907–1.506, *p* = 0.148). In the multivariate logistic regression analysis of 79 infants with complete data, adjusting for gestational age, the need for multiple surfactant doses remained a significant independent predictor of poor response (adjusted OR 0.244, 95% CI 0.086–0.693, *p* = 0.008). ELBW showed a trend toward poor response but did not reach statistical significance after adjustment (adjusted OR 0.281, 95% CI 0.062–1.268, *p* = 0.099).

## 4. Discussion

The aim of this study was to determine the contribution of genetic factors to NSAIDs’ response in the treatment of PDA in preterm neonates. Our findings demonstrate that genetic polymorphisms in CYP2C8*3 rs10509681, CYP2C9*2 rs1799853, *CYP2C9* rs2153628, and CYP2C9*3 rs1057910 were not significantly associated with NSAID treatment response in our cohort. Response failure was common in the current population (58%), with only 42% of infants achieving successful PDA closure, which is consistent with failure rates reported in other studies [21,27,28]. In a study by Dagle et al. evaluating the genetic variants associated with PDA, approximately 31% of infants with PDA underwent surgical ligation when the ductus remained open after medical treatment. In our cohort, however, the ligation rate was relatively lower (14%) and comparable to the 14% rate previously reported in a large cohort of more than 30,000 preterm infants [29,30]. Of note, other studies have described the decreasing use of surgical ligation, partly due to concerns regarding procedure-related morbidity, and the need for the development of selective treatment approaches to identify the infants most likely to benefit from ligation [31,32]. The majority of baseline characteristics were similar among responders and non-responders. In univariate analysis, extremely low birth weight and the need for multiple surfactant doses were significantly associated with poor response (*p* = 0.007 and *p* = 0.004, respectively).

In the multivariate analysis adjusting for gestational age, the need for multiple surfactant doses emerged as the strongest independent predictor of treatment failure, while ELBW showed a borderline association. The independent effect of surfactant requirements indicates that pulmonary disease severity may directly impact PDA responsiveness to NSAIDs, possibly through alterations in prostaglandin signaling or hemodynamic status. These findings have important clinical implications. Previous studies have demonstrated that persistent PDA and treatment failure are associated with prolonged respiratory support and adverse pulmonary outcomes, including bronchopulmonary dysplasia (BPD) [17,33]. This may be explained by increased pulmonary blood flow, particularly in infants requiring invasive ventilation for more than ten days [34,35,36,37]. These downstream consequences underscore the importance of identifying baseline predictors of treatment response to guide early therapeutic decisions. The independent association between higher surfactant needs and poor NSAID response suggests that infants with more severe respiratory disease at baseline may benefit from alternative treatment strategies or closer monitoring for treatment failure.

Gestational age emerged as a differing factor among groups in various studies [17,27,33]. Nevertheless, in the present study, no significant differences were observed in this regard even when analyzed as a continuous variable. After accounting for birth weight and disease severity, gestational age did not independently predict treatment response, suggesting that birth weight and pulmonary disease severity capture the maturity-related effects on NSAID response. This could also be explained by the small sample size, the fact that infants were recruited from a single center only, and the homogeneity of our population (85% extremely preterm at < 28 weeks).

To our knowledge, this is the first study to report on the associations between genetic variants and response to NSAIDs in infants with PDA in the Middle East region. Additionally, this is the first study to report on the frequency of the rs2153628 SNP within a population from the same region. Although other PGx results in neonates from the MENA region exist [38], they do not pertain to patients with PDA. Hence, this study contributes to the literature within this region and lays the groundwork for future investigations involving larger sample sizes. Moreover, the inclusion of clinical variables enabled the identification of non-genetic covariates that could result in medication response variations in preterm neonates. Despite identifying surfactant requirements as an independent predictor, most variations in response remain unexplained by clinical factors alone, supporting the hypothesis that genetic factors may play an important role in determining NSAID efficacy in this population.

This study has several limitations. First, the small sample size resulted in limited statistical power to detect genetic associations. This reflects the inherent challenge of recruiting infants for pharmacogenetic studies. Replication in larger, multi-center cohorts is needed to confirm our findings. Second, we examined only four polymorphisms in *CYP2C8* and *CYP2C9* genes, selected based on prior evidence of associations with NSAID outcomes. Other genetic variants potentially affecting prostaglandin signaling pathways (*PTGS1* [COX-1] and *PTGS2* [COX-2]) were not assessed due to resource constraints.

Furthermore, the retrospective design comes with certain limitations, particularly related to clinical data retrieval and completeness. In addition, survivorship bias, whereby only infants who survived and were discharged were included in the study, is an inherent limitation of our retrospective design with prospective sample collection and can limit the generalizability of our findings to the most critically ill infants. Nevertheless, given the rarity of eligible preterm infants and the need for long-term follow-up to identify sufficient cases, a retrospective design was the most practical approach and aligns with previous research with similar objectives. Also, only infants whose parents consented to buccal sampling consented to the use of de-identified electronic health record data for research. Therefore, differences in clinical data between enrolled and non-enrolled infants cannot be completely excluded. Finally, the DNA concentration obtained fell within the lower range, primarily due to challenges faced during the collection of buccal samples from infants. However, utilizing this method was the most practical approach to avoid the need for invasive techniques, which could have further reduced the sample size.

## 5. Conclusions

The findings indicate that carriers of CYP2C8*3 rs10509681, CYP2C9*2 rs1799853, *CYP2C9* rs2153628, and CYP2C9*3 rs1057910 did not show an association with variations in response to ibuprofen and indomethacin for PDA. In the analysis of non-genetic factors, the need for multiple surfactant doses was an independent predictor of poor response to treatment. Future studies with larger cohorts, diverse populations, and functional analyses are warranted to clarify potential pharmacogenetic influences and to explore the additional genetic and non-genetic factors that may contribute to treatment variability.

## Figures and Tables

**Table 1:** Characteristics of studied genetic variants.

Gene	SNP Name	Alleles	Phenotype
*CYP2C8*	(* 3) rs10509681	T > C	Decreased clearance
*CYP2C9*	(* 2) rs1799853	C > T	Decreased function
*CYP2C9*	rs2153628	A > G	Decreased clearance
*CYP2C9*	(* 3) rs1057910	A > C	No function

SNP: single-nucleotide polymorphism.

**Table 2:** Reagents and volumes used for the reaction mix for PCR.

Reaction Component	Volumes per Well (96-Well Plate)
DME (rs10509681, rs1799853, rs1057910)	
TaqMan Master Mix 2X	12.5 µL
20X Assay Working Stock	1.25 µL
Nuclease-Free Water	0 µL
DNA	11.25 µL
Total Volume Per Well	25 µL
SNP (rs2153628)	
TaqMan Master Mix 2×	5 µL
Genotyping Assay Mix 20×	0.5 µL
DNA	4.5 µL
Total Volume Per Well	10 µL

DME: drug metabolizing enzyme; SNP: single-nucleotide polymorphism; µL: microliter.

**Table 3:** The temperature, duration, and number of cycles used in each step of the PCR reaction.

Step	Temperature	Duration	Cycles
DME (rs10509681, rs1799853, rs1057910)			
AmpliTaq GoldR, UP, Enzyme Activation	95 °C	10 min	HOLD
Denaturation	92 °C	15 s	50
Annealing/Extension	60 °C	90 s	50
SNP (rs2153628)			
AmpliTaq GoldR, UP, Enzyme Activation	95 °C	10 min	HOLD
Denaturation	95 °C	15 s	40
Annealing/Extension	60 °C	1 min	40

DME: drug metabolizing enzyme; SNP: single-nucleotide polymorphism.

**Table 4:** Baseline characteristics.

Characteristic	Non-Responders (n = 50)	Responders (n = 36)	*p* Value
GA (weeks)—median (IQR)	25.1 (2.8)	26.3 (2.9)	0.148
Birth weight category—n (%)			0.033
VLBW	7 (14)	12 (33.3)	
ELBW	43 (86)	24 (66.7)	
Age on first day of treatment (days)—median (IQR)	8.5 (6.8)	8 (5.3)	0.298
Male sex—n (%)	29 (58)	19 (52.8)	0.63
Ethnicity—n (%)			0.589
Middle Eastern	28 (56)	22 (61.1)	
Asian	18 (36)	10 (27.8)	
Black	3 (6)	4 (11.1)	
White	1 (2)	0	
Delivery mode—n (%)			0.967
NVD	28 (56)	20 (55.6)	
CS	22 (44)	16 (44.4)	
Singleton pregnancy—n (%)	32 (64)	28 (77.8)	0.17
APGAR score at 1 min—median (IQR)	5.5 (3.8)	5 (4.3)	0.94
APGAR score at 5 min—median (IQR)	7.5 (1)	8 (3)	0.419
Chorioamnionitis—n (%)	6 (12)	2 (5.6)	0.31
Antenatal steroid use—n (%)	41 (82)	30 (83.3)	0.872
Surfactant use—n (%)	45 (90)	33 (91.7)	0.793
Surfactant doses—n (%)			0.01
1	10 (21.7)	17 (51.5)	
2	35 (76.1)	14 (42.4)	
3	1 (2.2)	2 (6.1)	
Resuscitation mode—n (%)			0.196
Noninvasive	7 (14)	9 (25)	
Invasive	43 (86)	27 (75)	
RDS severity—n (%)			0.798
Mild	13 (26)	8 (22.2)	
Moderate	17 (34)	11 (30.6)	
Severe	20 (40)	17 (47.2)	
Prior paracetamol use—n (%)	5 (10)	2 (5.6)	0.457
Respiratory support on first day of treatment—n (%)			0.119
Noninvasive	15 (30)	17 (47.2)	
CMV	32 (64)	15 (41.7)	
HFOV	3 (6)	4 (11.1)	
Pre-treatment serum creatinine—mean (SD)	56.9 (11.8)	53.9 (17.7)	0.418

APGAR: appearance, pulse, grimace, activity, and respiration; CMV: conventional mechanical ventilation; CS: cesarean section; ELBW: extremely low birth weight (<1000 g); GA: gestational age; HFOV: high-frequency oscillatory ventilation; IQR: interquartile range; n: number; NVD: normal vaginal delivery; RDS: respiratory distress syndrome; SD: standard deviation; VLBW: very low birth weight (<1500 g).

**Table 5:** Frequencies of genetic variants in *CYP2C8* and *CYP2C9* genes among study participants.

SNPs	All Patients	Non-Responders (n = 50)	Responders (n = 36)	*p* Value
CYP2C8*3 rs10509681				0.884
Wild type (TT)	36 (41.9)	20 (40)	16 (44)	
Heterozygous (TC)	39 (45.3)	23 (46)	16 (44)	
Homozygous (CC)	11 (12.8)	7 (14)	4 (11)	
CYP2C9*2 rs1799853				0.388
Missing (n = 3)				
Wild type (CC)	68 (81.9)	41 (82)	27 (75)	
Heterozygous (CT)	14 (16.9)	7 (14)	7 (19.4)	
Homozygous (TT)	1 (1.2)	0	1 (2.7)	
*CYP2C9* rs2153628				0.507
Missing (n = 23)				
Wild type (AA)	34 (39.5)	20 (40)	14 (38)	
Heterozygous (AG)	27 (31.4)	16 (32)	11 (30.5)	
Homozygous (GG)	2 (2.3)	2 (4)	0	
CYP2C9*3 rs1057910				0.928
Missing (n = 2)				
Wild type (AA)	72 (83)	41 (82)	31 (86)	
Heterozygous (AC)	12 (13.9)	7 (14)	5 (13.9)	
Values are presented as n (%).				

**Table 6:** Univariate and multivariate logistic regression of clinical variables and response to NSAID treatment.

Variable	Univariate			Multivariate		
	OR	95% CI	*p* Value	Adjusted OR	95% CI	*p* Value
GA	1.165	0.907–1.506	0.148	1.020	0.694–1.50	0.921
ELBW	0.326	0.098–0.695	0.007	0.281	0.062–1.268	0.099
Need for 2+ doses	0.261	0.084–0.625	0.004	0.244	0.086–0.693	0.008

CI: confidence interval; ELBW: extremely low birth weight; GA: gestational age, in weeks; OR: odds ratio.

## Data Availability

The original contributions presented in this study are included in the article. Further inquiries can be directed to the corresponding author.

## Supplementary Materials

The following supporting information can be downloaded at: https://www.mdpi.com/article/10.3390/jcdd13010049/s1, The STROBE Checklist for observational studies.

## References

[1] Nebert D.W. (1999). Pharmacogenetics and pharmacogenomics: Why is this relevant to the clinical geneticist?. Clin. Genet..

[2] Trivedi M.H. (2016). Right patient, right treatment, right time: Biosignatures and precision medicine in depression. World Psychiatry.

[3] van Schaik R.H. (2013). Clinical Application of Pharmacogenetics: Where are We Now?. Ejifcc.

[4] Van Driest S.L., McGregor T.L. (2013). Pharmacogenetics in clinical pediatrics: Challenges and strategies. Pers. Med..

[5] Zhao W., Leroux S., Biran V., Jacqz-Aigrain E. (2018). Developmental pharmacogenetics of CYP2C19 in neonates and young infants: Omeprazole as a probe drug. Br. J. Clin. Pharmacol..

[6] Sellmer A., Bjerre J.V., Schmidt M.R., McNamara P.J., Hjortdal V.E., Høst B., Bech B.H., Henriksen T.B. (2013). Morbidity and mortality in preterm neonates with patent ductus arteriosus on day 3. Arch. Dis. Child. Fetal Neonatal Ed..

[7] Hamrick S.E., Hansmann G. (2010). Patent ductus arteriosus of the preterm infant. Pediatrics.

[8] Backes C.H., Hill K.D., Shelton E.L., Slaughter J.L., Lewis T.R., Weisz D.E., Mah M.L., Bhombal S., Smith C.V., McNamara P.J. (2022). Patent Ductus Arteriosus: A Contemporary Perspective for the Pediatric and Adult Cardiac Care Provider. J. Am. Heart Assoc..

[9] Lee L.C., Tillett A., Tulloh R., Yates R., Kelsall W. (2006). Outcome following patent ductus arteriosus ligation in premature infants: A retrospective cohort analysis. BMC Pediatr..

[10] Park J., Yoon S.J., Han J., Song I.G., Lim J., Shin J.E., Eun H.S., Park K.I., Park M.S., Lee S.M. (2021). Patent ductus arteriosus treatment trends and associated morbidities in neonates. Sci. Rep..

[11] Sekar K.C., Corff K.E. (2008). Treatment of patent ductus arteriosus: Indomethacin or ibuprofen?. J. Perinatol..

[12] Thomas R.L., Parker G.C., Van Overmeire B., Aranda J.V. (2005). A meta-analysis of ibuprofen versus indomethacin for closure of patent ductus arteriosus. Eur. J. Pediatr..

[13] Ohlsson A., Walia R., Shah S.S. (2013). Ibuprofen for the treatment of patent ductus arteriosus in preterm and/or low birth weight infants. Cochrane Database Syst. Rev..

[14] Ohlsson A., Walia R., Shah S.S. (2020). Ibuprofen for the treatment of patent ductus arteriosus in preterm or low birth weight (or both) infants. Cochrane Database Syst. Rev..

[15] Evans P., O’Reilly D., Flyer J.N., Soll R., Mitra S. (2021). Indomethacin for symptomatic patent ductus arteriosus in preterm infants. Cochrane Database Syst. Rev..

[16] Weisz D.E., Mirea L., Rosenberg E., Jang M., Ly L., Church P.T., Kelly E., Kim S.J., Jain A., McNamara P.J. (2017). Association of Patent Ductus Arteriosus Ligation With Death or Neurodevelopmental Impairment Among Extremely Preterm Infants. JAMA Pediatr..

[17] Smith C.J., Ryckman K.K., Bahr T.M., Dagle J.M. (2017). Polymorphisms in CYP2C9 are associated with response to indomethacin among neonates with patent ductus arteriosus. Pediatr. Res..

[18] Blanco G., Martínez C., García-Martín E., Agúndez J.A.G. (2005). Cytochrome P450 Gene Polymorphisms and Variability in Response to NSAIDs. Clin. Res. Regul. Aff..

[19] Krasniqi V., Dimovski A., Domjanović I.K., Bilić I., Božina N. (2016). How polymorphisms of the cytochrome P450 genes affect ibuprofen and diclofenac metabolism and toxicity. Arh. Hig. Rada Toksikol..

[20] Lewis T., Benitz W.E., Smith P.B. (2019). Chapter 12—Neonatal Pharmacogenetics. Infectious Disease and Pharmacology.

[21] Chen X., Chen Y., Xiao T., Dong X., Lu Y., Qian Y., Wang H., Zhou W. (2022). CYP2C9*3 Increases the Ibuprofen Response of Hemodynamically Significant Patent Ductus Arteriosus in the Infants with Gestational Age of More Than 30 Weeks. Phenomics.

[22] HCY 150 mm Medical Disposable Sampling Tube for Saliva Collecting. https://icleanswabs.com/.

[23] PROMEGA Maxwell^®^ 16 DNA Purification Kits Technical Manual. https://worldwide.promega.com/resources/protocols/technical-manuals/0/maxwell-16-dna-purification-kits-protocol/.

[24] Thermo Fisher Scientific NanoDrop™ 2000/2000c Spectrophotometers. https://www.thermofisher.com/order/catalog/product/ND-2000.

[25] Thermo Fisher Scientific TaqMan™ Drug Metabolism Genotyping Assay. https://www.thermofisher.com/order/catalog/product/4362691?SID=srch-srp-4362691.

[26] Thermo Fisher Scientific TaqMan™ SNP Genotyping Assay, Human. https://www.thermofisher.com/order/catalog/product/4331349?SID=srch-srp-4331349.

[27] Sallmon H., Aydin T., Hort S., Kubinski A., Bode C., Klippstein T., Endesfelder S., Bührer C., Koehne P. (2019). Vascular endothelial growth factor polymorphism rs2010963 status does not affect patent ductus arteriosus incidence or cyclooxygenase inhibitor treatment success in preterm infants. Cardiol. Young.

[28] Dagle J.M., Lepp N.T., Cooper M.E., Schaa K.L., Kelsey K.J., Orr K.L., Caprau D., Zimmerman C.R., Steffen K.M., Johnson K.J. (2009). Determination of genetic predisposition to patent ductus arteriosus in preterm infants. Pediatrics.

[29] Weinberg J.G., Evans F.J., Burns K.M., Pearson G.D., Kaltman J.R. (2016). Surgical ligation of patent ductus arteriosus in premature infants: Trends and practice variation. Cardiol. Young.

[30] Dagle J.M., Ryckman K.K., Spracklen C.N., Momany A.M., Cotten C.M., Levy J., Page G.P., Bell E.F., Carlo W.A., Shankaran S. (2019). Genetic variants associated with patent ductus arteriosus in extremely preterm infants. J. Perinatol..

[31] Reese J., Scott T.A., Patrick S.W. (2018). Changing patterns of patent ductus arteriosus surgical ligation in the United States. Semin. Perinatol..

[32] Ibrahim T., Abdul Haium A.A., Tapawan S.J., Dela Puerta R., Allen J.C., Chandran S., Chua M.C., Rajadurai V.S. (2020). Selective Treatment of PDA in High-Risk VLBW Infants With Birth Weight ≤800 g or <27 Weeks and Short-Term Outcome: A Cohort Study. Front. Pediatr..

[33] Rooney S.R., Shelton E.L., Aka I., Shaffer C.M., Clyman R.I., Dagle J.M., Ryckman K., Lewis T.R., Reese J., Van Driest S.L. (2019). CYP2C9*2 is associated with indomethacin treatment failure for patent ductus arteriosus. Pharmacogenomics.

[34] El-Khuffash A., Mullaly R., McNamara P.J. (2023). Patent ductus arteriosus, bronchopulmonary dysplasia and pulmonary hypertension—A complex conundrum with many phenotypes?. Pediatr. Res..

[35] Clyman R.I., Hills N.K. (2023). Patent ductus arteriosus (PDA) and pulmonary morbidity: Can early targeted pharmacologic PDA treatment decrease the risk of bronchopulmonary dysplasia?. Semin. Perinatol..

[36] Clyman R.I., Kaempf J., Liebowitz M., Erdeve O., Bulbul A., Håkansson S., Lindqvist J., Farooqi A., Katheria A., Sauberan J. (2021). Prolonged Tracheal Intubation and the Association Between Patent Ductus Arteriosus and Bronchopulmonary Dysplasia: A Secondary Analysis of the PDA-TOLERATE trial. J. Pediatr..

[37] Clyman R.I., Hills N.K. (2020). The effect of prolonged tracheal intubation on the association between patent ductus arteriosus and bronchopulmonary dysplasia (grades 2 and 3). J. Perinatol..

[38] Sridharan K., Al Jufairi M., Al Ansari E., Jasim A., Eltayeb Diab D., Al Marzooq R., Al Madhoob A. (2021). Evaluation of urinary acetaminophen metabolites and its association with the genetic polymorphisms of the metabolising enzymes, and serum acetaminophen concentrations in preterm neonates with patent ductus arteriosus. Xenobiotica.

