# Supplementary material for: Evaluation of CYP2C8 and CYP2C9 Polymorphisms in Neonates with Patent Ductus Arteriosus Treated with Ibuprofen or Indomethacin: A Retrospective Cohort Study
Source: J Cardiovasc Dev Dis. 2026 Jan 15;13(1):49. doi: 10.3390/jcdd13010049 (PMC12842244; doi:10.3390/jcdd13010049)
Supplement: Supplementary file 1 [file jcdd-13-00049-s001.zip › jcdd-4012631-supplementary.pdf]

STROBE Statement—Checklist of items that should be included in reports of ***cohort studies***

	Item No	Recommendation	Section
**Title and abstract**	1	(*a*) Indicate the study's design with a commonly used term in the title or the abstract	Design is indicated in the abstract
		(*b*) Provide in the abstract an informative and balanced summary of what was done and what was found	Abstract summarized the study and covered background, methods, results and conclusion.
**Introduction**			
Background/rationale	2	Explain the scientific background and rationale for the investigation being reported	Introduction, 1st to last paragraph
Objectives	3	State specific objectives, including any prespecified hypotheses	Introduction, last paragraph
**Methods**			
Study design	4	Present key elements of study design early in the paper	Method, section 2.1
Setting	5	Describe the setting, locations, and relevant dates, including periods of recruitment, exposure, follow-up, and data collection	Method, section 2.1
Participants	6	(*a*) Give the eligibility criteria, and the sources and methods of selection of participants. Describe methods of follow-up	Method, section 2.1
		(*b*) For matched studies, give matching criteria and number of exposed and unexposed	NA
Variables	7	Clearly define all outcomes, exposures, predictors, potential confounders, and effect modifiers. Give diagnostic criteria, if applicable	Method, section 2.1
Data sources/ measurement	8*	For each variable of interest, give sources of data and details of methods of assessment (measurement). Describe comparability of assessment methods if there is more than one group	Method, section 2.1-2.4
Bias	9	Describe any efforts to address potential sources of bias	Method, section 2.5
Study size	10	Explain how the study size was arrived at	Method, section 2.5
Quantitative variables	11	Explain how quantitative variables were handled in the analyses. If applicable, describe which groupings were chosen and why	Method, section 2.1 and 2.5
Statistical methods	12	(*a*) Describe all statistical methods, including those used to control for confounding	Method, section 2.5
		(*b*) Describe any methods used to examine subgroups and interactions	NA
		(*c*) Explain how missing data were addressed	Paragraph below Table 5. Only complete data were included in the multivariate regression.
		(*d*) If applicable, explain how loss to follow-up was addressed	NA

		(*e*) Describe any sensitivity analyses	NA
**Results**			
Participants	13*	(a) Report numbers of individuals at each stage of study—eg numbers potentially eligible, examined for eligibility, confirmed eligible, included in the study, completing follow-up, and analysed	Results, Figure 1
		(b) Give reasons for non-participation at each stage	Results, Figure 1
		(c) Consider use of a flow diagram	Results, Figure 1
Descriptive data	14*	(a) Give characteristics of study participants (eg demographic, clinical, social) and information on exposures and potential confounders	Results, Table 4
		(b) Indicate number of participants with missing data for each variable of interest	Results, missing surfactant doses described in the paragraph under Table 5.
		(c) Summarise follow-up time (eg, average and total amount)	Results, first paragraph
Outcome data	15*	Report numbers of outcome events or summary measures over time	Results, first paragraph
Main results	16	(*a*) Give unadjusted estimates and, if applicable, confounder-adjusted estimates and their precision (eg, 95% confidence interval). Make clear which confounders were adjusted for and why they were included	Results, Table 6
		(*b*) Report category boundaries when continuous variables were categorized	Results, Table 4 footnotes
		(*c*) If relevant, consider translating estimates of relative risk into absolute risk for a meaningful time period	NA
Other analyses	17	Report other analyses done—eg analyses of subgroups and interactions, and sensitivity analyses	NA
**Discussion**			
Key results	18	Summarise key results with reference to study objectives	Discussion, first paragraph
Limitations	19	Discuss limitations of the study, taking into account sources of potential bias or imprecision. Discuss both direction and magnitude of any potential bias	Discussion, first paragraph
Interpretation	20	Give a cautious overall interpretation of results considering objectives, limitations, multiplicity of analyses, results from similar studies, and other relevant evidence	Discussion, second to last and last paragraph
Generalisability	21	Discuss the generalisability (external validity) of the study results	Discussion, third paragraph (i.e. single center, homogenous sample)
**Other information**			
Funding	22	Give the source of funding and the role of the funders for the present study and, if applicable, for the original study on which the present article is based	Funding statement provided

*Give information separately for exposed and unexposed groups.

Note: An Explanation and Elaboration article discusses each checklist item and gives methodological background and published examples of transparent reporting. The STROBE checklist is best used in conjunction with this article (freely available on the Web sites of PLoS Medicine at http://www.plosmedicine.org/, Annals of Internal Medicine at http://www.annals.org/, and Epidemiology at http://www.epidem.com/). Information on the STROBE Initiative is available at http://www.strobe-statement.org.
